# Fargesin ameliorates osteoarthritis via macrophage reprogramming by downregulating MAPK and NF-κB pathways

**DOI:** 10.1186/s13075-021-02512-z

**Published:** 2021-05-14

**Authors:** Jiansen Lu, Hongbo Zhang, Jianying Pan, Zhiqiang Hu, Liangliang Liu, Yanli Liu, Xiao Yu, Xiaochun Bai, Daozhang Cai, Haiyan Zhang

**Affiliations:** 1 Department of Joint Surgery, Center for Orthopaedic Surgery, The Third Affiliated Hospital of Southern Medical University, Guangzhou, China; 2 Department of Orthopedics, Orthopedic Hospital of Guangdong Province, Academy of Orthopedics Guangdong Province, The Third Affiliated Hospital of Southern Medical University, Guangzhou, China; 3 The Third School of Clinical Medicine, Southern Medical University, Guangzhou, China; 4 Guangdong Provincial Key Laboratory of Bone and Joint Degeneration Diseases, Guangzhou, China; 5 Department of Immunology, School of Basic Medical Sciences, Southern Medical University, Guangzhou, China

**Keywords:** Osteoarthritis, Fargesin, Macrophage, Mitogen-activated protein kinase pathway, Nuclear factor kappa-B pathway

## Abstract

**Background:**

To investigate the role and regulatory mechanisms of fargesin, one of the main components of *Magnolia fargesii*, in macrophage reprogramming and crosstalk across cartilage and synovium during osteoarthritis (OA) development.

**Methods:**

Ten-week-old male C57BL/6 mice were randomized and assigned to vehicle, collagenase-induced OA (CIOA), or CIOA with intra-articular fargesin treatment groups. Articular cartilage degeneration was evaluated using the Osteoarthritis Research Society International (OARSI) score. Immunostaining and western blot analyses were conducted to detect relative protein. Raw264.7 cells were treated with LPS or IL-4 to investigate the role of polarized macrophages. ADTC5 cells were treated with IL-1β and conditioned medium was collected to investigate the crosstalk between chondrocytes and macrophages.

**Results:**

Fargesin attenuated articular cartilage degeneration and synovitis, resulting in substantially lower Osteoarthritis Research Society International (OARSI) and synovitis scores. In particular, significantly increased M2 polarization and decreased M1 polarization in synovial macrophages were found in fargesin-treated CIOA mice compared to controls. This was accompanied by downregulation of IL-6 and IL-1β and upregulation of IL-10 in serum. Conditioned medium (CM) from M1 macrophages treated with fargesin reduced the expression of matrix metalloproteinase-13, RUNX2, and type X collagen and increased Col2a1 and SOX9 in OA chondrocytes, but fargesin alone did not affect chondrocyte catabolic processes. Moreover, fargesin exerted protective effects by suppressing p38/ERK MAPK and p65/NF-κB signaling.

**Conclusions:**

This study showed that fargesin switched the polarized phenotypes of macrophages from M1 to M2 subtypes and prevented cartilage degeneration partially by downregulating p38/ERK MAPK and p65/NF-κB signaling. Targeting macrophage reprogramming or blocking the crosstalk between macrophages and chondrocytes in early OA may be an effective preventive strategy.

## Introduction

Osteoarthritis (OA) is a joint disease that mainly afflicts weight-bearing joints and has a high incidence and rate of disability in aging populations. It is predicted to affect nearly 67 million people in the USA by 2030 [1–3]. The global prevalence of OA is higher in women and increases with age [4]. Some projections suggest that opioid dispensing and associated costs in OA will substantially increase between 2015 and 2030 [5].

OA is characterized by progressive degradation of articular cartilage, subchondral bone remodeling, vascular invasion, osteophyte formation, and synovial inflammation [6]. Synovial inflammation acts as a key factor during OA pathogenesis, which is associated with increased cartilage degeneration, increased serious joint symptoms, elevated radiographic grades, and decreased mobility [7–11]. A large multicenter study showed synovial inflammation or effusion in 46% of people with symptomatic knee OA [12]. Normal synovium is an immune organ that consists of macrophages and fibroblast-like synoviocytes and lymphocytes. Cartilage fragments, fibronectin, aggrecan, and intracellular protein necrotic cells act as danger-associated molecular patterns (DAMPs) during OA [13, 14]. Macrophages can be activated and polarized (M1 or M2) by DAMPs and then produce cytokines and chemokines. M1 macrophages secrete large amounts of pro-inflammatory cytokines, such as tumor necrosis factor-α (TNF-α), interleukin (IL)-1, IL-6, and IL-12. M2 macrophages maintain their anti-inflammatory activity and secrete some anti-inflammatory cytokines, such as IL-4, IL-10, and IL-13 [15, 16]. Recent studies have shown that macrophage polarization plays an important role in OA [17]. However, macrophage polarization regulation is poorly understood in OA.

Mitogen-activated protein kinases (MAPKs) are crucial regulators of cellular pathology and physiology and include ERK, p38, and JNK MAPK subfamilies [18–20]. Recent studies have shown that MAPK plays a crucial role in chondrogenic differentiation [21, 22]. However, it is unknown whether MAPK activation has a beneficial or detrimental effect on the regulation of macrophage polarization and OA amelioration. The nuclear factor kappa-light-chain-enhancer of activated B cells (NF-κB) protein complex plays important roles in several biological processes, including proliferation, differentiation, aging, cell survival, apoptosis, inflammation, and immune responses. The NF-κB signaling pathway is critical for the induction of various inflammation-related cytokines and mediators, including inducible nitric oxide synthase (iNOS), matrix metalloproteinase (MMP) proteins, TNF-α, and IL-1β [23, 24]. However, the molecular mechanisms involved in macrophage polarization remain unknown.

Fargesin is one of the main components of *Magnolia fargesii* and has traditionally been used to treat sinusitis and inflammation. Moreover, fargesin’s anti-inflammatory effects can inhibit NF-ĸB signaling and reduce nitric oxide in a variety of cell types [25–27]. A recent study has shown that fargesin can inhibit murine malignancy by regulating the P38/MAPK signaling pathway [28]. However, the exact mechanism describing potential contributions of fargesin in macrophage polarization regulation during OA progression is largely unknown.

The present study found that fargesin increased M2 polarization and decreased M1 polarization in synovial macrophages in collagenase-induced osteoarthritis (CIOA), a high synovial activation OA model. It also attenuated articular cartilage degeneration and synovitis. Protective effects of fargesin in OA cartilage were evident through partial suppression of p38/ERK MAPK and p65/NF-κB signaling. Thus, targeting macrophage reprogramming or blocking crosstalk between macrophages and chondrocytes represent novel therapeutic strategies for OA treatment.

## Materials and methods

### Animals

All animal experiments were approved by the Southern Medical University Animal Care and Use Committee (SMUL2021014). Ten-week-old male C57/BL6 mice (23–30 g) were purchased from the Laboratory Animal Centre of Southern Medical University (No. 44002100018098).

### Mouse model

Ten-week-old male C57/BL6 mice (*n* = 48) were subjected to intra-articular injection collagenase to induce CIOA as previously described [29]. Briefly, 1 U of collagenase (C0773; Sigma-Aldrich, St. Louis, MO, USA) was injected into the right knee joint twice on alternate days. Only skin of the right knee joint was resected in the sham-operated group. Some mice from the CIOA group were treated with fargesin (5, 10, or 20 mg/kg, 10 μL, *n* = 36; PHL82537, Sigma, USA), while others were treated with vehicle (20% DMSO dissolved in saline) by intra-articular injection twice a week for 1, 3, or 6 weeks (10 μL, *n* = 12). Then, 1, 3, or 6 weeks after operation, mice from each group were sacrificed for collection of the right knee joint (Additional file 1: Figure S1A). Articular cartilage degeneration was quantified using the Osteoarthritis Research Society International (OARSI) scoring system. H&E staining was used to evaluate synovial activation by scoring synovial lining cell thickness (0–3), as previously described [29].. Then, the sum of medial and lateral joint compartments was determined (0–6).

### Cells

Raw264.7 macrophages from the American Type Culture Collection (ATCC, USA) were grown in Dulbecco’s modified Eagle’s medium (DMEM) with high glucose (4.5 g/L; Gibco, USA), containing 100 U/mL penicillin, 100 mg/mL streptomycin sulfate (Life Technologies, USA), and 10% FBS (Gibco, USA). The pre-chondrocyte cell line ATDC5 (Tsukuba, Japan) was maintained in DMEM/F12 (Gibco, USA), containing 100 U/mL penicillin, 100 mg/mL streptomycin sulfate, 5% FBS, and 1× ITS universal culture supplement premix reagent (BD Biosciences).

### Drug treatment

Raw264.7 cells were treated with lipopolysaccharides (LPS; 100 ng/mL; Peprotech, USA) for 24 h to induce M1-like macrophages and with IL-4 (20 ng/mL; Peprotech, USA) for 24 h to induce M2-like macrophages. Fargesin (10, 20, and 40 μM) was administered in Raw264.7 cells for 24 h. Cells treated with 0.1% DMSO served as a control in vitro. Media samples were then collected and kept at − 20 °C until further analysis for cytokine determination.

### Primary chondrocyte culture

Rib cartilage from newborn mice (24–72 h) was dissected under a stereo light microscope to harvest primary chondrocytes. After trypsin digestion for 30 min, primary chondrocytes were separated, purified, and digested in 0.1% collagenase type II (Sigma, USA) with 10% FBS, 100 U/mL penicillin, and 100 mg/mL streptomycin sulfate at 37 °C for 4–6 h. Primary chondrocytes were resuspended and seeded in a 24-well plate and cultured in DMEM/F12 with 10% FBS, 100 U/mL penicillin, and 100 mg/mL streptomycin sulfate at 37 °C with 5% CO_2_.

### Co-culture

Raw264.7 cells were treated with LPS (100 ng/mL) for 24 h to induce M1-like macrophages. Vehicle or fargesin (20 μM) was administered in Raw264.7 cells induced with LPS for 24 h. Supernatants were collected and co-cultured with ATDC5 for 24 h in a 24-well plate (500 μl supernatants without dilution). In addition, ATDC5 cells were treated with IL-1β (100 ng/mL) for 24 h to induce OA-like cells. Vehicle or fargesin (20 μM) was administered in ATDC5 cells induced with IL-1β for 24 h. Supernatants were collected and co-cultured with Raw264.7 for 24 h in a 24-well plate (500 μl supernatants without dilution).

### ELISA

Serum and cell supernatants were analyzed using mouse IL-6, IL-10, and IL-1β ELISA kit (#E-EL-M0044c, #E-EL-M0046c, and #E-EL-M0037c; Elabscience Biotechnology). ELISA analysis was performed according to the manufacturer’s instructions.

### Quantitative reverse transcription-polymerase chain reaction

Total RNA was extracted from tissues or cultured cells using TRIzol reagent (Invitrogen, Thermo Fisher Scientific, Waltham, MA, USA), as previously described [24]. cDNA was reverse transcribed using TaKaRa reverse transcription reagents (TaKaRa Bio Inc., Shiga, Japan) and PCR was performed using Real-Time PCR Mix (TaKaRa) on a light cycler (Roche, Basel, Switzerland) with the following primers: ColX (forward primer 5′-AAA GCT TAC CCA GCA GTA GG-3′ and reverse primer 5′-ACG TAC TCA GAG GAG TAG AG-3′), MMP13 (forward primer 5′-CTT CTT GTT GAG CTG GA CTC-3′ and reverse primer 5′-CTG TGG AGG TCA CTG TAG ACT-3′), Runx2 (forward primer 5′-TCC CCG GGA ACC AAG AAG GCA-3′ and reverse primer 5′-AGG GAG GGC CGT GGG TTC TG-3′), and GAPDH (forward primer 5′-AGG TCG GTG TGA ACG GAT TTG-3′ and reverse primer 5′-TGT AGA CCA TGT AGT TGA GGT CA-3′). The glyceraldehyde 3-phosphate dehydrogenase gene was used as an endogenous control to normalize the differences in the amount of total RNA.

### Preparation of decalcified sections, histochemistry, immunostaining, and immunohistochemistry

Freshly dissected mouse knee joints were fixed in 4% paraformaldehyde for 24 h at 4 °C and decalcified in 14% EDTA (pH 7.4) for 30 days at 25 °C. Tissues were embedded in paraffin and sectioned continuously (3-μm thick). Safranin O/Fast Green staining was performed as previously described [24]. For immunohistochemistry and immunofluorescence, sections were soaked in citrate buffer (10 mM citric acid, pH 6.0) for 16 h at 62 °C or treated with 0.1 mg/mL proteinase K (Sigma-Aldrich) for 15 min at 37 °C to unmask antigens after deparaffinization and rehydration. For immunohistochemistry, 3% hydrogen peroxide solution was added for 15 min. Sections were blocked with 10% sheep serum at 37 °C for 1–2 h and incubated with primary antibodies (in 1% BSA, 0.1% Triton X-100) at 4 °C overnight. Sections were then incubated with secondary antibodies at 37 °C for 1 h. Furthermore, 3,3′-diaminobenzidine was used to observe chromogen and hematoxylin during counterstaining. For immunofluorescence, species-matched antibodies labeled with Alexa Fluor 488 and 594 or horseradish peroxidase (HRP) were used (1:100 in 1% BSA) as previously described [24]. Nuclei were labeled with 4′,6-diamidino-2-phenylindole (Thermo) before imaging.

### Western blotting

Lysis buffer was prepared with 10% glycerol, 2% sodium dodecyl sulfate, 10 mM dithiothreitol, 10 mM Tris–HCl (pH 6.8), 1 mM phenylmethylsulfonyl fluoride, and 10% β-mercaptoethanol. Tissues and cells were lysed at 98 °C for 10 min. Samples were separated with SDS-PAGE for 90 min, blotted onto nitrocellulose membranes for 1 h, and blocked with 5% milk at 37 °C for 1–2 h. Then, membranes were incubated with primary antibodies (in 5% BSA, 0.2% NaN3) at 4 °C overnight. Samples were incubated with secondary antibodies at 37 °C for 1 h.

### Antibodies

The following antibodies were used in this study: rabbit anti-Col X [1:100 for immunohistochemistry (IHC); Abcam, USA; ab58632], rabbit anti-MMP-13 (1:100 for IHC; Abcam, USA; ab39012), rabbit anti-RUNX2 (1:100 for IHC; Abclone, Australia; A2851), rabbit anti-mannose receptor (1:100 for IF; Acam, USA; ab64693), rabbit anti-iNOS (1:100 for IF; Abclone, Australia; A3200), anti-rabbit IgG light chain (1:100 for IHC; Abbkine, USA; A25022), HRP-labeled goat anti-rabbit IgG H&L (1:1000 for western blots, 1:100 for IHC; Jackson Immuno Research, USA; 111-035-003), HRP-labeled goat anti-mouse IgG H&L (1:3000 for western blots; Jackson Immuno Research; 115–035-003), Alexa Fluor 594-labeled goat anti-mouse IgG H&L [1:500 for immunofluorescent labeling (I); Abcam; ab150120], and Alexa Fluor 488-labeled goat anti-rabbit IgG H&L (1:500 for IF; Abcam; ab150077).

### Materials

Dimethyl-sulphoxide (DMSO) was obtained from Sigma-Aldrich (St Louis, MO, USA). Cell Counting Kit-8 (CCK-8) was purchased from Keygen Biotech (Nanjing, China). Fargesin (purity > 98%) was dissolved in 100% DMSO (400 mg/mL). Then, fargesin storage solution dissolved in saline.

### Statistical analysis

All experiments were performed three times. Data were represented as mean ± SD using SPSS version 19.0 software (SPSS, USA). Curve analysis was performed using GraphPad Prism 5.0 (USA). Data in each group were analyzed using an unpaired, two-tailed Student’s *t*-test. The level of significance was set at *p* < 0.05.

## Results

### Fargesin attenuates cartilage damage and synovitis in CIOA

**Fig. 1:**
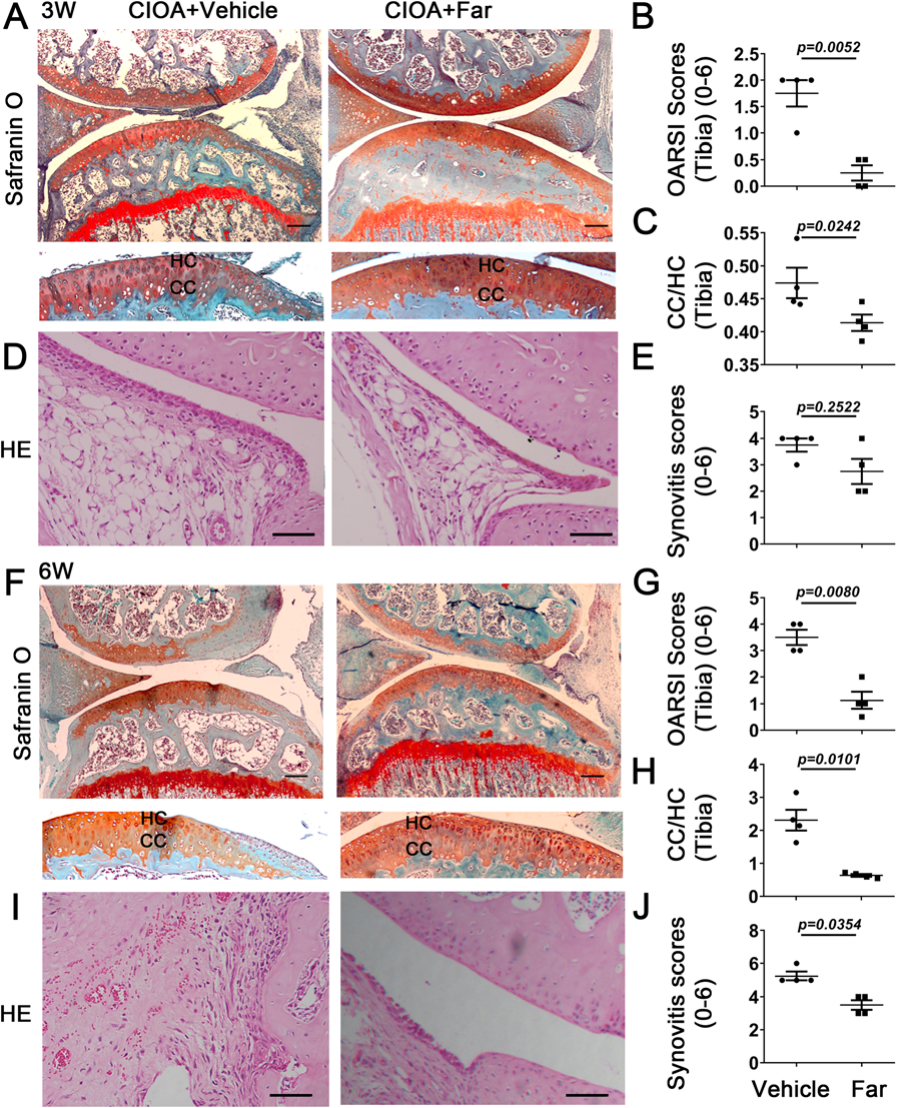
Fargesin attenuates cartilage damage and synovitis in CIOA. (**a**, **f**) Cartilage degradation assessed by Safranin O and Fast Green staining. Dotted lines represent tide line. Scale bar: 100 μm (top) and 40 μm (bottom). (**b**, **g**) OARSI score was evaluated in CIOA mice treated with vehicle or fargesin. (**c**, **h**) Cartilage thickness was evaluated using ratio between calcified cartilage (CC) and hyaline cartilage (HC). (**d**, **i**) H&E staining in CIOA mice treated with vehicle or fargesin. Scale bar: 100 μm. (**e**, **j**) Synovitis score quantification in CIOA mouse synovium treated with vehicle or fargesin (*n* = 4)

To investigate the potential role of fargesin in OA, intra-articular injection of fargesin was performed in CIOA mice. After comparative analysis of the fargesin effect at several concentrations (5, 10, or 20 mg/kg) in OA progression, 10-mg/kg fargesin was used in subsequent animal experiments (Additional file 1: Figure S1B). Interestingly, fargesin administration showed retention of proteoglycans and decreased calcified cartilage thickness compared to control mice both 3 and 6 weeks after intra-articular injection of collagenase, as confirmed by the OARSI score (Fig. 1a, b, c, f, g, h). No significant differences in the OARSI score were present between fargesin and control mice 1 week after intra-articular injection (data not shown). Synovial inflammation was further investigated in both mouse groups. Although fargesin treatment demonstrated a slight decrease in synovial hyperplasia, no significant differences in the knee synovitis score were present between fargesin and control mice 3 weeks after intra-articular injection (Fig. 1d, i). However, the synovitis score was significantly reduced in fargesin-treated mice 6 weeks after intra-articular injection (Fig. 1e, j). These findings demonstrated that fargesin prevented OA progression to cartilage damage and synovial inflammation.

### Fargesin acts as a potent polarizer towards M2 macrophages

**Fig. 2:**
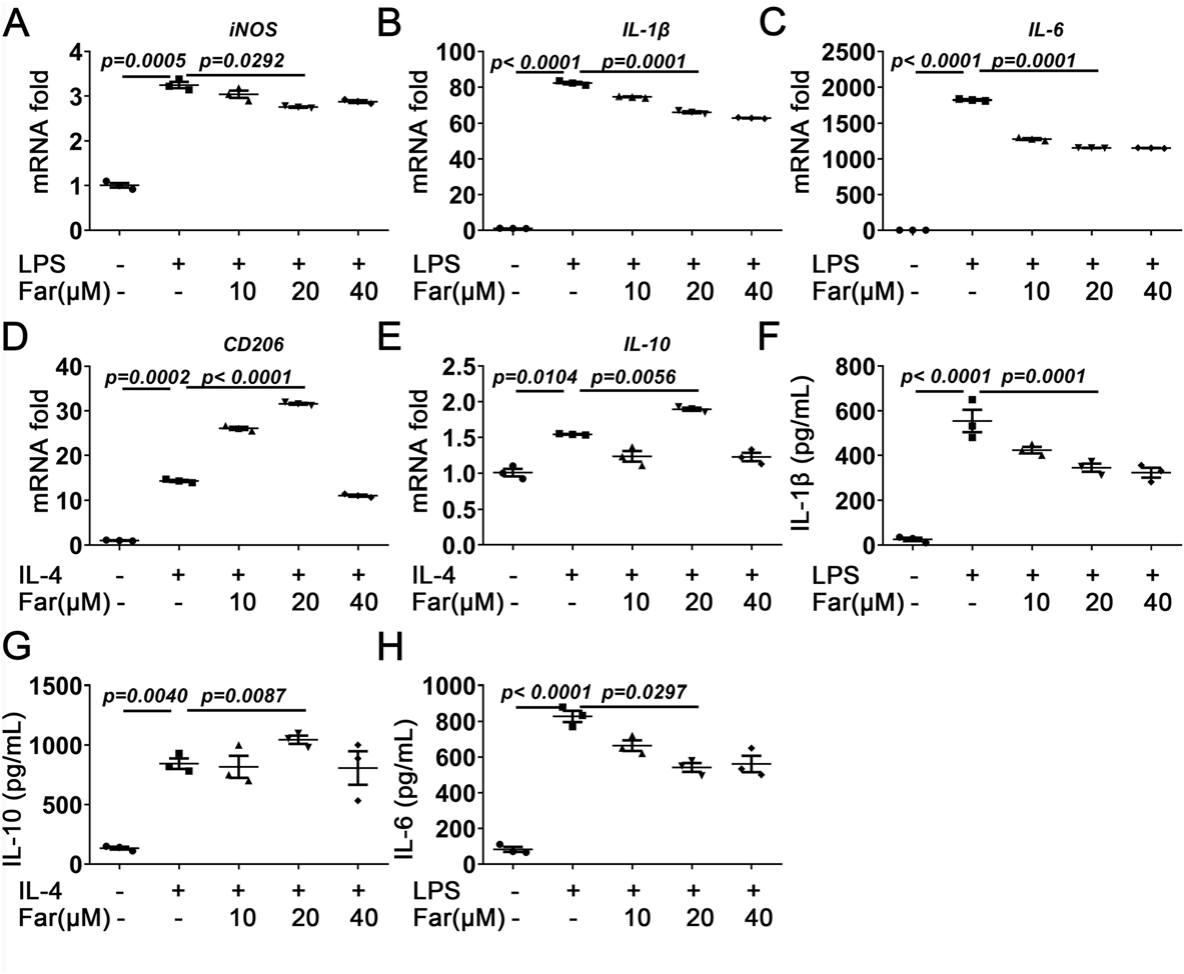
Fargesin acts as a potent polarizer towards M2 macrophages. **a**–**c** Quantitative PCR analysis of iNOS, IL-1β, and IL-6 in LPS-induced Raw264.7 cells treated with or without fargesin. **d**, **e** Quantitative PCR analysis of CD206, and IL-10 in IL-4-stimulated Raw264.7 cells treated with or without fargesin. **f** After ASC plasmid transfection and treatment with or without fargesin in LPS-induced Raw264.7 cells, the release of IL-1β still needed ATP-induced inflammasome, and then cell supernatants were detected by ELISA. **h**, **g** ELISA results for IL-6 and IL-10 levels in Raw264.7 cell supernatant treated with LPS or IL-4 and co-treated with or without fargesin (*n* = 3). ASC, apoptosis-associated speck

Our previous study demonstrated a strong effect of macrophage polarization in synovial inflammation and OA development [24]. Although fargesin has been shown to exert anti-inflammatory effects and traditionally has been used to treat sinusitis, the exact mechanism describing potential fargesin contributions to macrophage polarization regulation during OA progression is largely unknown. The effect of fargesin on macrophage polarization was determined at concentrations ranging between 10 and 20 μM. Fargesin significantly affects cell viability at a concentration of only 80 μM for 24 h compared to the untreated control (Additional file 2: Supplementary Figure S2A and B). Initially, the M1/M2 macrophage phenotype marker genes induced by LPS (M1 inducer) and IL-4 (M2 inducer) were analyzed using RT-PCR. After fargesin exposure, IL-4-induced CD206 mRNA level was further upregulated and LPS-induced iNOS was significantly downregulated in Raw264.7 cells, indicating a potential role of fargesin in macrophage reprogramming (Fig. 2a, d). Fargesin decreased pro-inflammatory factors (iNOS, IL-1β, and IL-6) in a concentration-dependent manner. However, the effect of fargesin on anti-inflammatory factors (CD206 and IL-10) did not absolutely depend on the concentration. The anti-inflammatory effects were present at a concentration of 20 μM. The anti-inflammatory effects of fargesin may be more complex. According to the CCK-8 assay, Raw 264.7 cells treaded with IL-4 and fargesin (40 μM) for 24 h affected cell survival (Additional file 2: Figure S2C). Furthermore, fargesin mounted anti-inflammatory cytokine IL-10 and inhibited pro-inflammatory cytokines IL-1β and IL-6 secreted by polarized macrophages. Production levels of IL-10, IL-1β, and IL-6 were confirmed by ELISA (Fig. 2b, c, e–h). Interestingly, optimum fargesin concentration in macrophage polarization was 20 μM. The effect of fargesin in macrophages was confirmed in THP-1 cells (Additional file 3: Figure S3). Together, these data suggested that fargesin played a role in macrophage reprogramming to M2 subtype.

### Fargesin enhances M2 macrophage polarization and mounts anti-inflammatory cytokines in OA synovium and serum

**Fig. 3:**
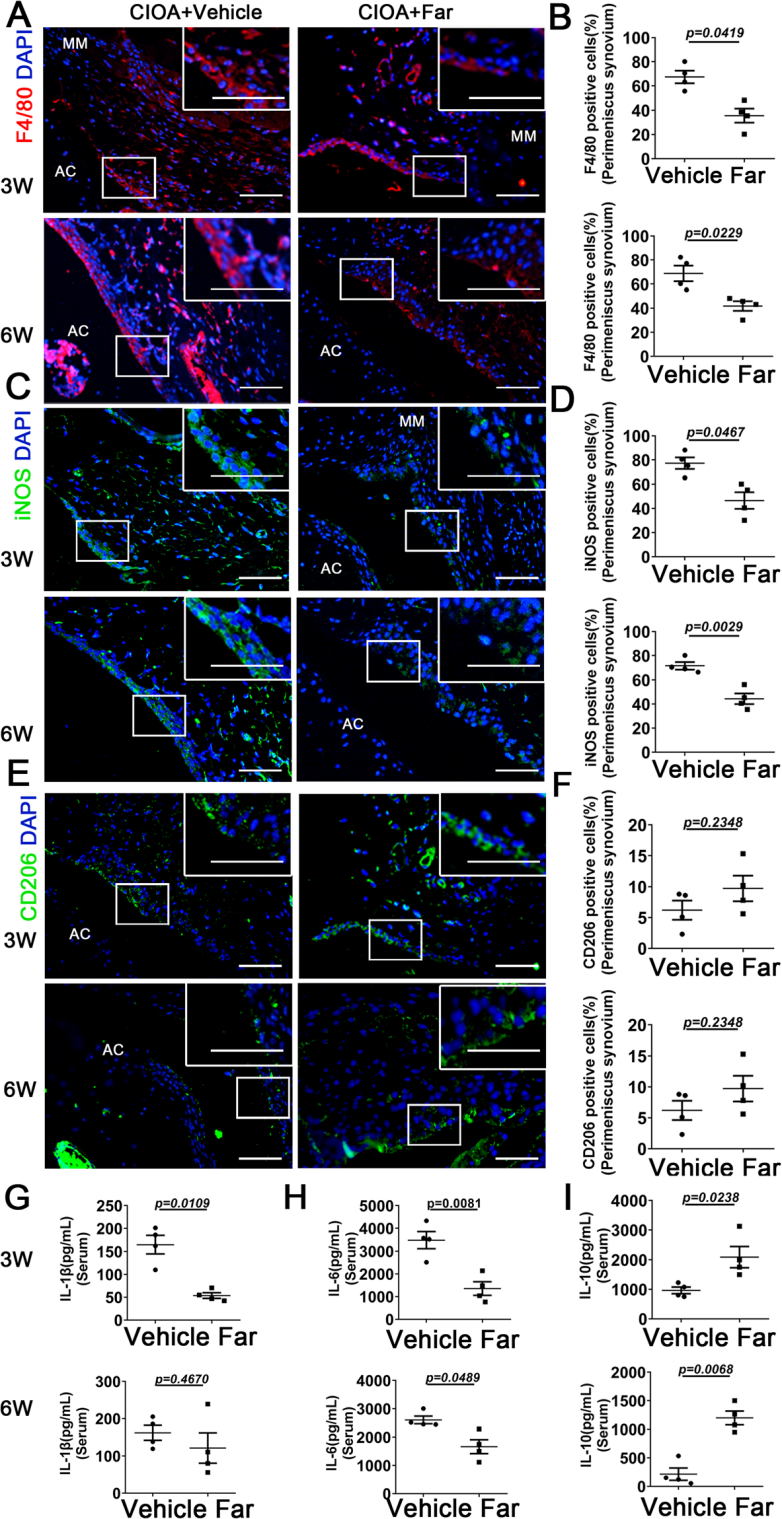
Fargesin enhances M2 macrophage polarization and mounts anti-inflammatory cytokines in OA synovium and serum. (**a**-**f**) Immunostaining and quantitative analysis of cells positive for F4/80 (**a**, **b**), iNOS (**c**, **d**), and CD206 (**e**, **f**) in CIOA mice treated with vehicle or fargesin three and six weeks after intra-articular injection collagenase. Scale bar: 50 μm. Higher magnification is shown on the right top. Scale bar: 100 μm. (**g**-**i**) ELISA results for IL-1β, IL-6, and IL-10 levels in CIOA mouse serum treated with vehicle or fargesin three and six weeks after intra-articular injection collagenase (*n* = 4). AC, articular cartilage; MM, medial meniscus

Phenotypic characterization of macrophages was determined in OA synovial mouse tissue treated with fargesin. Compared to controls, a marked reduction in F4/80 (macrophage marker)-positive cells was detected in fargesin-treated OA mice, together with a significant decrease in iNOS (M1-like macrophage marker)-positive cells (Fig. 3a–d). In contrast, the proportion of cells positive for M2-like macrophage marker CD206 in fargesin-treated OA synovium was significantly increased predominantly in the intimal lining layer 3 weeks, but not 6 weeks, after CIOA surgery (Fig. 3e, f). Consistent with the in vitro study, downregulation of IL-6 and upregulation of IL-10 were detected in the serum of fargesin-treated CIOA mice compared to controls both 3 and 6 weeks after CIOA administration. However, a slight decrease in IL-1β in fargesin-treated CIOA mouse serum was not statistically significant compared to controls 6 weeks after intra-articular injection (Fig. 3g–i). These results suggested that fargesin played a crucial role in OA development by enhancing M2 macrophage polarization and mounting anti-inflammatory cytokines.

### Fargesin reprograms macrophages from M1 to M2 subtype via p38/ERK MAPK and p65/NF-κB pathways

**Fig. 4:**
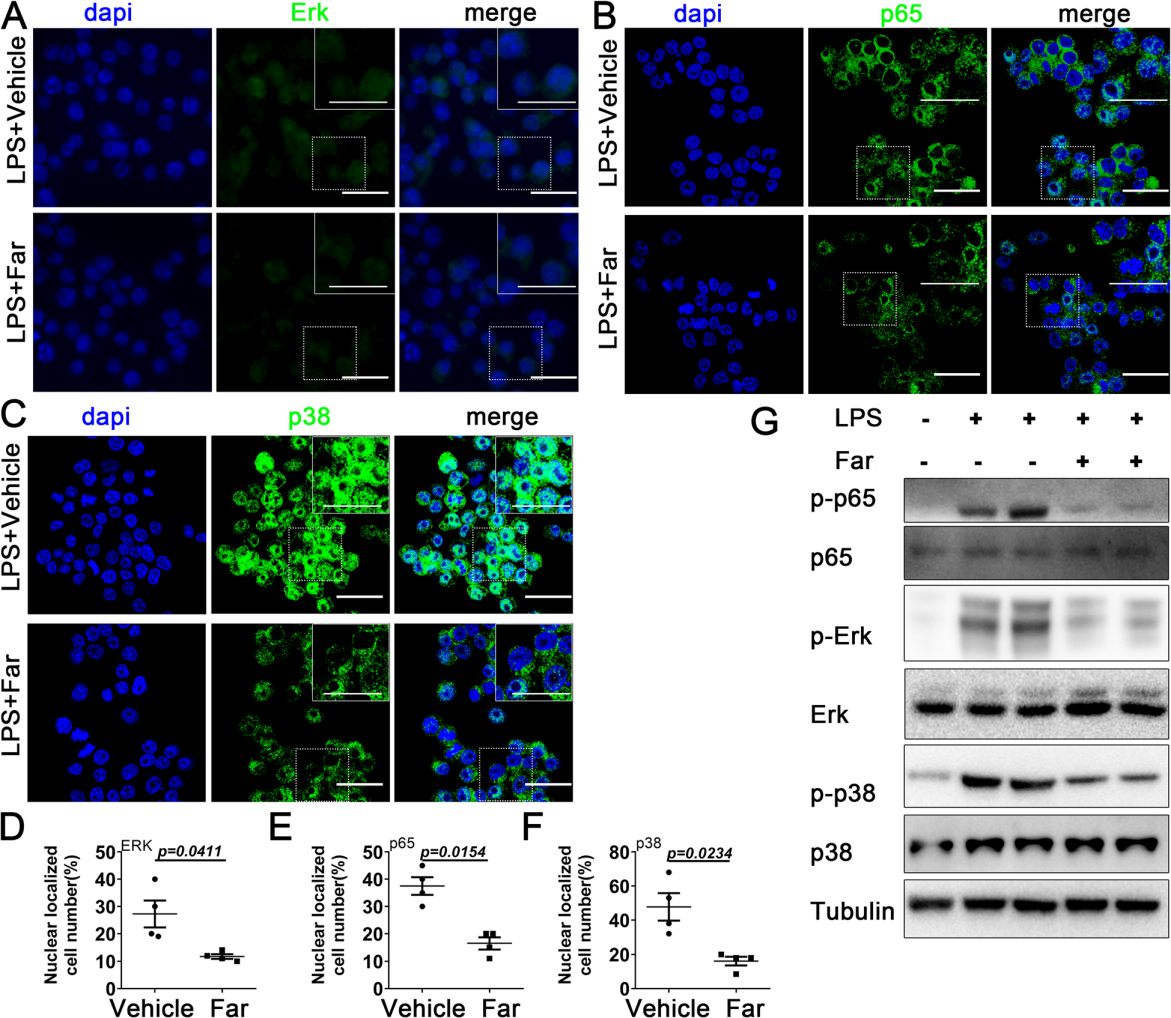
Fargesin reprograms macrophages from M1 to M2 subtype via p38/ERK MAPK and p65/NF-κB pathways. **a**–**f** Immunostaining and quantitative analysis of nuclear localized cells for ERK, p38, and p65 in Raw264.7 cells treated with LPS (1 μg/mL) for 30 min after administering vehicle or fargesin for 3 h (*n* = 4). Scale bar, 50 μm. Higher magnification is shown on the top right. Scale bar, 100 μm. **g** Western blot results for p-p65, p65, p-ERK, ERK, p-p38, and p38 in Raw264.7 cells treated with LPS and co-treated with or without fargesin (*n* = 4)

The mechanism of macrophage polarization regulation is quite complex and little is known about its regulation during the pathogenesis and progression of OA. This study sought to identify fargesin-derived pathways responsible for macrophage reprogramming during OA. It was determined that fargesin treatment can rescue the phosphorylation of p38/ERK MAPK and p65 NFκB signaling, which were activated by LPS during M1 polarization (Fig. 4a–f, Additional file 4: Figure S4). This indicated that fargesin blocked M1 macrophage polarization by inhibiting the MAPK and NFκB pathways. Moreover, downregulated expression of p-p38, p-ERK, and p-p65 after fargesin treatment was confirmed in M1 macrophage (Fig. 4g). These findings suggested that fargesin had the ability to block M1 macrophage polarization and convert M1 polarized cells into M2 cells via p38/ERK MAPK and p65 NFκB, resulting in OA development amelioration.

### Fargesin protects chondrocytes from catabolism via paracrine macrophage mechanism

**Fig. 5:**
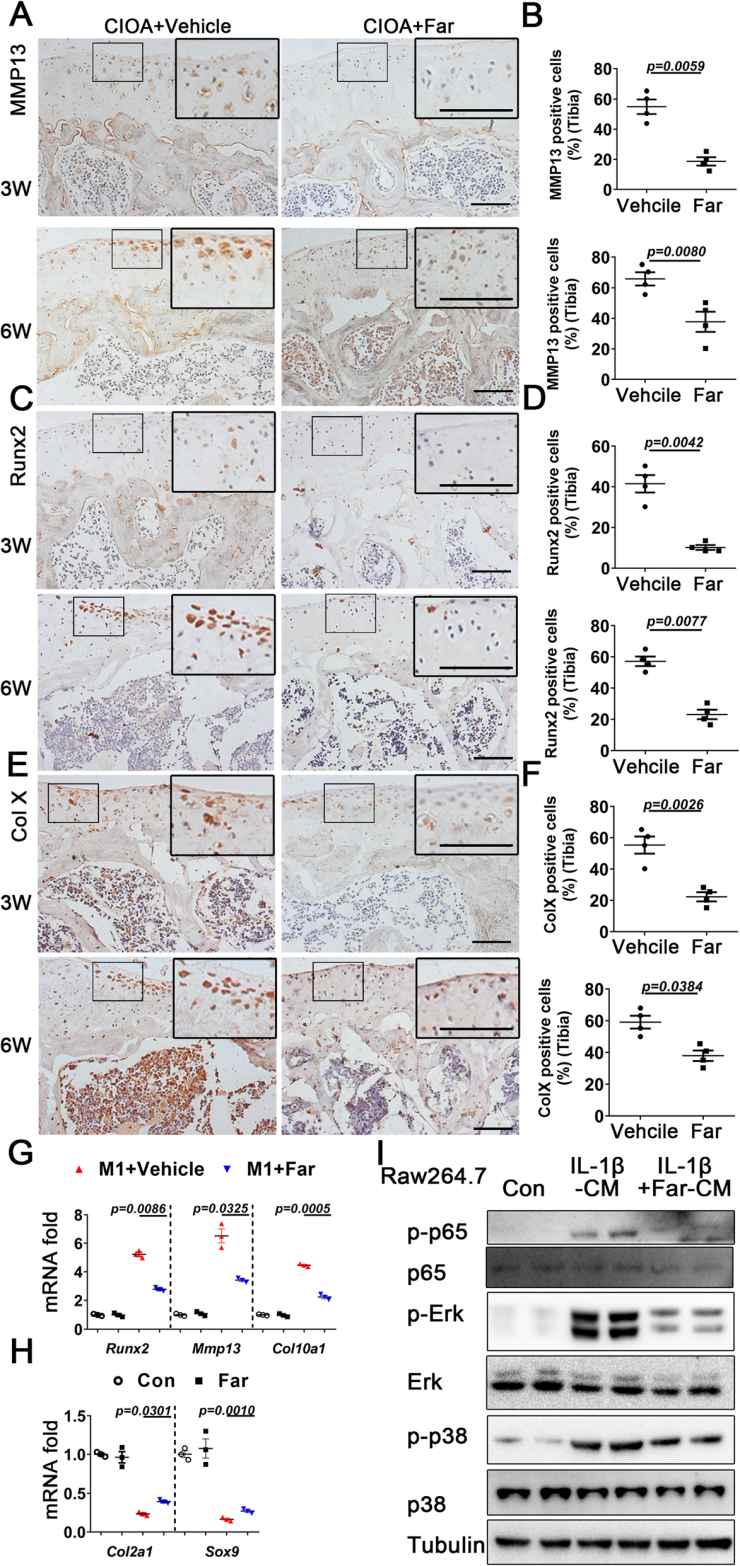
Fargesin protects chondrocytes from catabolism via paracrine macrophage mechanism. **a–f** Immunostaining and quantitative analysis of cells positive for MMP13, RUNX2, and Col X in CIOA mice treated with vehicle or fargesin 3 and 6 weeks after intra-articular injection of collagenase (*n* = 4). Scale bar, 50 μm. Higher magnification is shown on the top right. Scale bar, 100 μm. **g**, **h** Raw264.7 cells were treated with vehicle (served as Con) or fargesin (served as Far) for 24 h and conditioned medium (CM) were collected. LPS-induced Raw264.7 cells were co-treated with vehicle (served as M1+Vehicle) or fargesin (served as M1+Far) for 24 h and CM were collected. Quantitative PCR analysis of MMP13, RUNX2, Col X, Col2, and Sox9 in ATDC5 cells treated with different CM from Raw 264.7 cells (respectively Con, Far, M1+vehicle, and M1+Far) for 24 h. **i** ATDC5 cells were treated with vehicle (served as Con) for 24 h and CM were collected. IL-1β-induced ATDC5 cells were co-treated with vehicle (served as IL-1β-CM) or fargesin (served as IL-1β-CM+Far) for 24 h and CM were collected. Western blot results for p-p65, p65, p-ERK, ERK, p-p38, and p38 in Raw264.7 cells treated with different CM (respectively Con, IL-1β-CM, and IL-1β-CM+Far) from ATDC5 (*n* = 3). AC, articular cartilage; MM, medial meniscus

Compelling data suggest that in addition to the autocrine mechanism, paracrine interactions between macrophages and chondrocytes serve as an additional mechanism that plays a central role during initiation and development of OA. MMP13 expression in fargesin-treated mouse cartilage was dramatically reduced compared to that of controls. Furthermore, RUNX2 (master transcription factor for pre-hypertrophic differentiation) and ColX (marker for hypertrophic chondrocytes) were also downregulated in articular cartilage after fargesin treatment (Fig. 5a–f). In order to explore the mechanism of fargesin in chondrocytes, ATDC5 cells were treated with or without fargesin after stimulation with insulin, transferrin, selenium, and conditioned medium (CM) from the M1 or M2 macrophage culture. Under basal conditions in chondrocytes, fargesin alone did not affect chondrocyte catabolic processes. However, fargesin had protective effects on chondrocytes in an inflammatory microenvironment, along with decreasing catabolic markers (MMP13, RUNX2, and ColX) and increasing chondrogenic markers (Col2 and Sox9), which showed no significant changes when treated by fargesin alone (Fig. 5g, h). Importantly, fargesin blocked the crosstalk between chondrocytes and macrophages. P38/ERK MAPK and p65 NFκB signaling pathways were activated by IL-1β-treated chondrocyte CM. Fargesin eventually reversed these phenotypic changes (Fig. 5i). Our previous results showed that the promoting macrophage M1 polarization activated by mTORC1 signaling leads to exacerbation of experimental OA partially through the secretion of R-spondin-2(Rspo2). Interestingly, p-S6, Rspo2, and β-catenin were inhibited by fargesin treatment in CIOA mice, indicating that fargesin may regulate mTORC1 and β-catenin signaling (Additional file 5: Figure S5). Taken together, these data suggested that fargesin reduces catabolic factors in chondrocytes and attenuates cartilage degeneration via paracrine macrophage mechanisms.

## Discussion

**Fig. 6:**
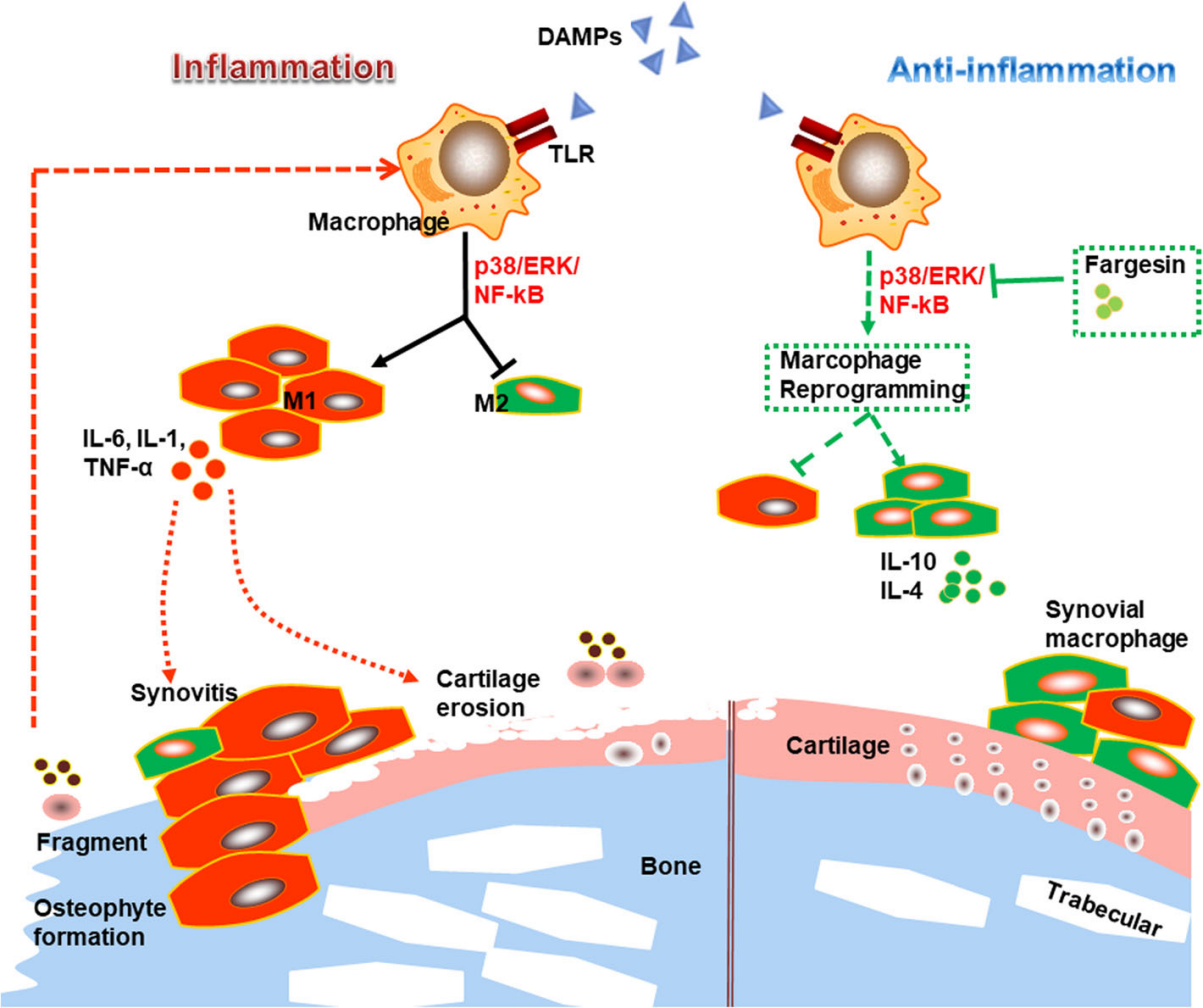
Positive feedback regulation model of macrophage activation via p38/ERK MAPK and p65/NF-κB signaling during CIOA development. Fargesin inhibits p38/ERK MAPK and p65/NF-κB activation to reprogram macrophages from M1 to M2 and mediates crosstalk between activated macrophages and apoptotic chondrocytes to prevent CIOA pathogenesis and progression

This study established the essential role of fargesin in the pathogenesis and progression of OA. It also proposed a pathway in which fargesin suppresses p38/ERK/NF-κB signaling in synovial tissues to reprogram macrophage polarization by converting M1 polarized cells into M2 cells and consequently ameliorates articular cartilage degeneration and OA synovitis (Fig. 6). Macrophage transformation reprogramming from M1 to M2 subtype or blocking paracrine interactions between macrophages and chondrocytes are thus potential therapeutic targets for OA treatment.

Accumulating evidence demonstrates that the imbalance of M1/M2 macrophage polarization plays an essential role in OA [30]. Our previous study demonstrated that M1- and not M2-polarized macrophages accumulate in human and mouse OA synovial tissue [29]. M1 macrophage polarization enhances the release of inflammatory factors, such as IL-6, IL-1β, MMPs, and TNF-α, resulting in promotion of nerve growth factor expression and causing OA pain. M2 macrophage polarization increases the release of anti-inflammatory factors, such as IL-10 and IL-4, and identifies the critical role of synovial M1 and M2 macrophages in the development of OA [31, 32]. However, macrophage depletion with both M1 and M2 subtypes has a confused effect on OA progression. These studies indicated that the failure of transformation from M1 to M2 subtype, more than the quantity of activated macrophages, may be a key link in the progression of OA. Fargesin is one of the main components of *Magnolia fargesii* and has traditionally been used to treat sinusitis and inflammation [26]. However, the role of fargesin in OA is largely unknown. The present study showed that fargesin attenuated cartilage damage and synovitis in CIOA mice. Moreover, fargesin had the ability to convert M1 polarized macrophages into M2 macrophages and mounting anti-inflammatory cytokines during OA development. However, low concentration of fargesin (10 and 20 μM) enhanced M2 polarized macrophages, but high concentration did not significantly enhanced M2 polarization, indicating that the potential clinical applications of fargesin will require strict dose control so that effective concentrations can be achieved in target tissues. DMSO has known anti-oxidant and anti-inflammatory properties, which may increase the anti-inflammatory effect of fargesin. In our present results, only a slight improvement without statistically significant difference was observed with vehicle (20% DMSO in saline) treatment compared to saline only treatment (Additional file 6: Figure S6). Fargesin may attenuate OA progression partially by preventing pathological M1 macrophage polarization and accumulation of compensatory M2 macrophages, indicating that fargesin mediated macrophage reprogramming in OA. However, IL-1β in fargesin-treated CIOA mouse serum and CD206 in fargesin-treated OA synovium were statistically significantly different compared to controls 6 weeks after surgery, suggesting that the role of fargesin in anti-inflammation was effective in the early stages of OA.

Recent studies have suggested that paracrine interactions between macrophages and chondrocytes provide another mechanism for the initiation and development of OA. Activated synovial macrophages showed that enhanced inflammation mediated the chondrocyte microenvironment and promoted MMP accumulation, which resulted in degradation of extracellular matrix (ECM) [33–35]. Consistently, ECM degradation acted as a DAMP, stimulating macrophage activation and increasing synovial inflammation, which resulted in positive feedback regulation of inflammation and cartilage degradation. Samavedi et al. [36] demonstrated that chondrocytes co-cultured with macrophage activation expressed significantly enhanced inflammatory factors, resulting in ECM degradation. Similarly, chondrocytes promoted various inflammatory factors in co-cultured macrophages, suggesting that cross-talk between macrophages and chondrocytes contributed to OA development. Inhibiting this positive feedback regulation of inflammation and cartilage degradation may attenuate OA progression. The present study found that intra-articular injection of fargesin did not only decrease the inflammatory factors in CIOA mouse serum, but also had a protective effect on cartilage by downregulating MMP13, RUNX2, and ColX expression and increasing Col2a1 and SOX9. Interestingly, fargesin also suppressed chondrocyte catabolism that is upregulated by polarized macrophages. However, fargesin alone did not affect chondrocyte catabolic processes. These results strongly suggested that fargesin may protect chondrocytes by mediating paracrine interactions between macrophages and chondrocytes.

Emerging evidence highlights the vital role of p38/ERK MAPK and p65/NF-κB signaling in macrophage activation and cartilage degradation, which promote pathogenesis and progression of OA [37, 38]. A recent study has shown an enhanced production of IL-6 and TNF-α in human synovial fibroblasts via activated ERK, p38, and JNK signaling pathways [39]. Kim et al. demonstrated that inhibition of the p38 MAPK signaling pathway suppresses apoptosis in human OA chondrocytes [40]. Chen et al. showed that blocking NF-κB suppresses IL-1β-induced expression of inflammatory cytokines in human OA chondrocytes, demonstrating a protective effect in mouse OA models [41]. Moreover, studies have shown that NF-κB and MAPK activation is involved in LPS-induced iNOS expression and macrophage polarization switch. The present study identified a major role of p38/ERK MAPK and p65/NF-κB signaling in crosstalk between activated macrophages and apoptotic chondrocytes. Consistent with previous research, the present study demonstrated that p38/ERK MAPK and p65/NF-κB signaling was activated in LPS-induced M1 polarization and highly expressed in synovial tissues during OA development [42, 43]. Importantly, fargesin treatment did not only inhibit upregulation of p38/ERK MAPK and p65/NF-κB signaling in vivo and in vitro, but also reversed the phenotypic changes in macrophages via IL-1β-treated chondrocyte CM. This further confirmed that fargesin can inhibit the crosstalk between activated macrophages and apoptotic chondrocytes via p38/ERK MAPK and p65/NF-κB signaling during OA.

In conclusion, these findings broaden the potential clinical applications of fargesin. Fargesin-attenuated OA progression occurred by targeting macrophage reprogramming in the early stages of OA. Fargesin switched the polarized phenotypes of macrophages from M1 to M2 subtypes and prevented cartilage degeneration partially by downregulating p38/ERK MAPK and p65/NF-κB signaling. Targeting macrophage reprogramming or blocking the crosstalk between macrophages and chondrocytes in early OA may be an effective preventive strategy.

## Conclusions

In summary, this study established the essential role of fargesin in pathogenesis and progression of OA. Fargesin switched the polarized phenotypes of macrophages from M1 to M2 subtypes and prevented cartilage degeneration partially by downregulating p38/ERK MAPK and p65/NF-κB signaling. Fully understanding the signaling pathways and targeting macrophage transformation reprogramming or blocking paracrine interactions between macrophages and chondrocytes may be effective preventive strategies for OA development.

## Supplementary Information

**Additional file 1: Figure S1.** Optimal fargesin dose screening in CIOA mice. (A) Drug treatment was administered at each stage. Ten-week-old mice were subjected to CIOA and treated with vehicle or fargesin. (B) Safranin O and Fast Green staining sagittal views of tibial medial cartilage 3 weeks after intra-articular injection of collagenase: sham, CIOA treated with vehicle, and CIOA treated with 5, 10, and 20 mg/kg fargesin. Scale bar: 100 μm. (C and D) OARSI score was evaluated in CIOA mice treated with vehicle or fargesin in both tibia and femur (0–12).

**Additional file 2: Figure S2.** Fargesin effects on cell viability in ATDC5 and Raw264.7 cells. (A and B) Cells were cultured with various concentrations of fargesin (0–80 μM) for 24 h and then evaluated using Cell Counting Kit-8 assay. (C) Raw264.7 cells were co-cultured with IL-4 (20 ng/mL) and various concentrations of fargesin (0–40 μM) for 24 h and then evaluated using Cell Counting Kit-8 assay. OD, optical density (*n* = 3).

**Additional file 3: Figure S3.** Fargesin acts as a potent polarizer towards M2 macrophages in THP-1 cells. (A–C) Quantitative PCR analysis of iNOS, IL-6, and CD206 in THP-1 cells treated with LPS or IL-4 and co-treated with vehicle or fargesin (n = 3).

**Additional file 4: Figure S4.** Fargesin treatment inhibits p65 nuclear translocation in LPS-induced Raw264.7 cell. (A and B) Immunostaining and quantitative analysis of nuclear localized cell for p65 in Raw264.7 cells treated with LPS (1 μg/mL) for 30 min after treated with vehicle or fargesin for 3 h. And control (Con) was treated without LPS (n = 3). Scale bar: 50 μm. Higher magnification is shown on the top right. Scale bar: 100 μm.

**Additional file 5: Figure S5.** Fargesin attenuates p-S6, Rspo2 and β-catenin expression in synovial tissue of CIOA mice. (A-F) Immunostaining and quantitative analysis of cells positive for F4/80, p-S6 (A and B), Rspo2 (C and D), and β-catenin (E and F) in CIOA mice treated with vehicle or fargesin 3 weeks after intra-articular injection of collagenase. Scale bar: 50 μm. Higher magnification is shown on the top right. Scale bar: 100 μm. AC, articular cartilage; MM, medial meniscus.

**Additional file 6: Figure S6.** No significant improvement is observed in vehicle treatment compared to saline treatment 3 weeks after intra-articular injection of collagenase. (A) Cartilage degradation assessed by Safranin O and Fast Green staining. Dotted lines represent tide line. Scale bar: 50 μm (top). (B) OARSI score was evaluated in CIOA mice treated with saline or vehicle. (C-F) Immunostaining and quantitative analysis of cells positive for CD206 (C and D) and iNOS (E and F) in CIOA mice treated with saline or vehicle 3 weeks after intra-articular injection of collagenase. Scale bar: 50 μm. Scale bar: 25 μm. AC, articular cartilage; MM, medial meniscus. (*n* = 3).

## Data Availability

All data generated or analyzed during this study are included in this published article and its additional files.
