# Supplementary figures and images for: Fargesin ameliorates osteoarthritis via macrophage reprogramming by downregulating MAPK and NF-κB pathways
Source: Arthritis Res Ther. 2021 May 14;23:142. doi: 10.1186/s13075-021-02512-z (PMC8120707; doi:10.1186/s13075-021-02512-z)

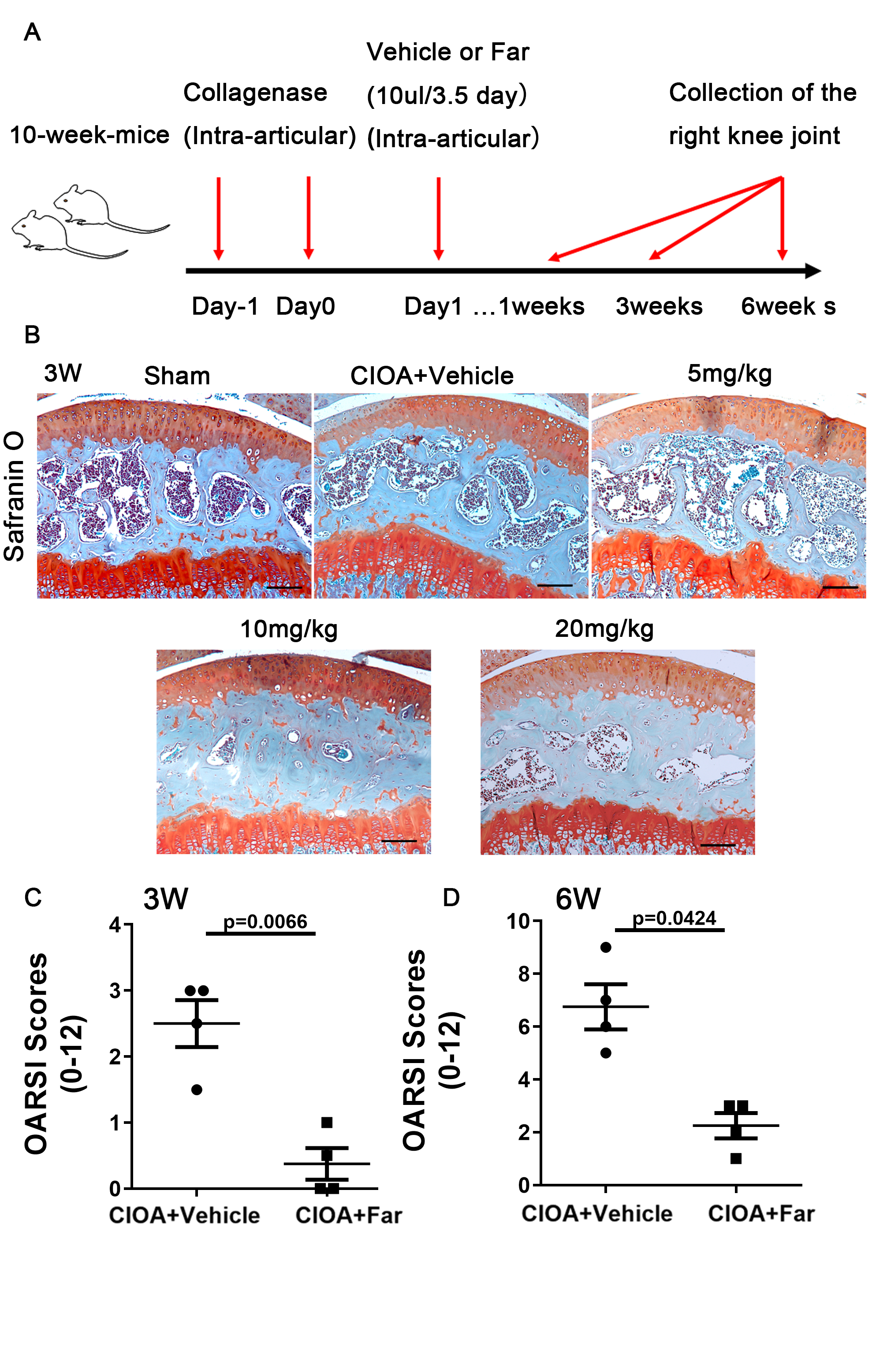

Supplement: Supplementary file 1 — Additional file 1: Figure S1. Optimal fargesin dose screening in CIOA mice. (A) Drug treatment was administered at each stage. Ten-week-old mice were subjected to CIOA and treated with vehicle or fargesin. (B) Safranin O and Fast Green staining sagittal views of tibial medial cartilage 3 weeks after intra-articular injection of collagenase: sham, CIOA treated with vehicle, and CIOA treated with 5, 10, and 20 mg/kg fargesin. Scale bar: 100 μm. (C and D) OARSI score was evaluated in CIOA mice treated with vehicle or fargesin in both tibia and femur (0–12). [file 13075_2021_2512_MOESM1_ESM.tif]

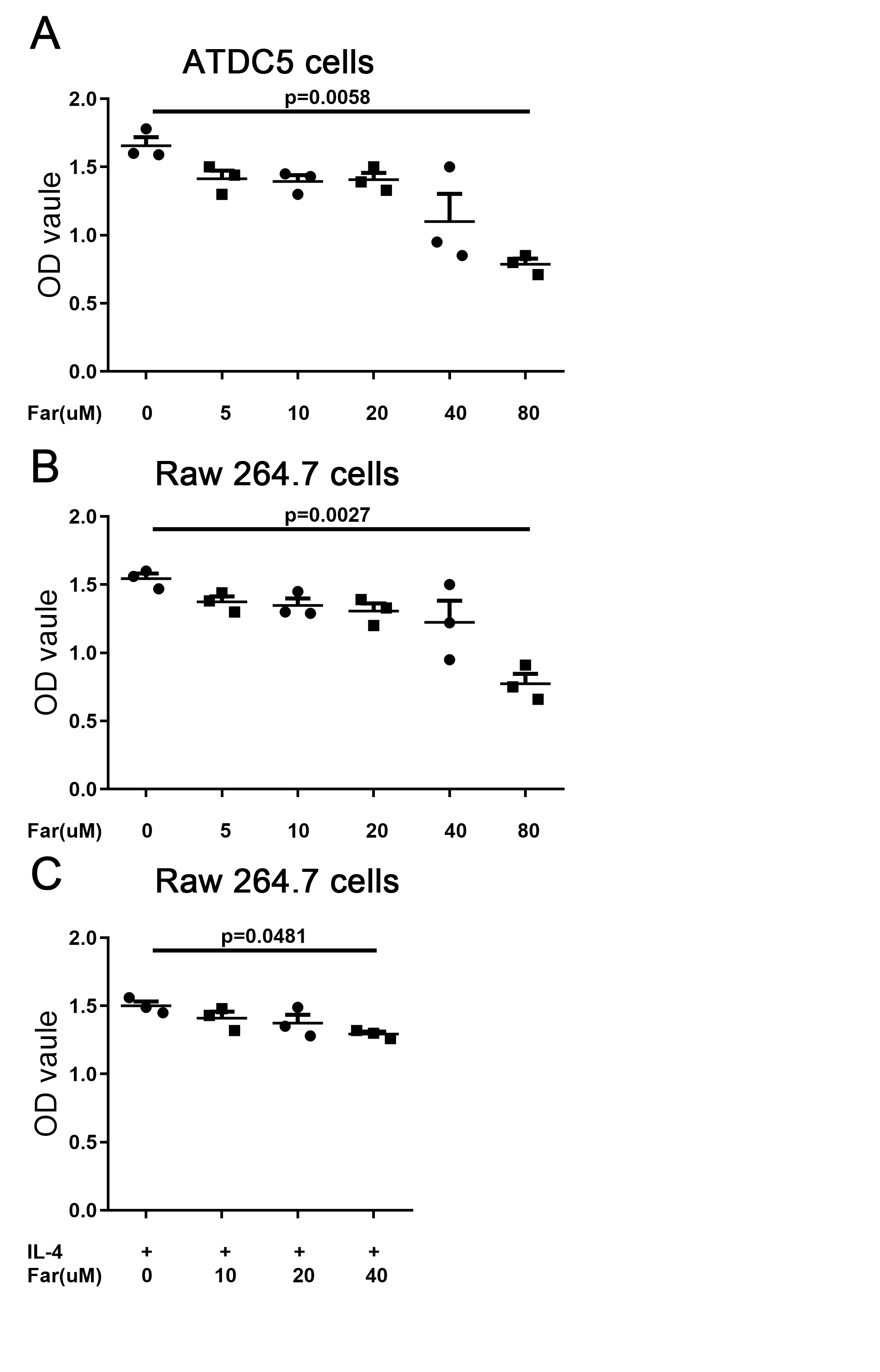

Supplement: Supplementary file 2 — Additional file 2: Figure S2. Fargesin effects on cell viability in ATDC5 and Raw264.7 cells. (A and B) Cells were cultured with various concentrations of fargesin (0–80 μM) for 24 h and then evaluated using Cell Counting Kit-8 assay. (C) Raw264.7 cells were co-cultured with IL-4 (20 ng/mL) and various concentrations of fargesin (0–40 μM) for 24 h and then evaluated using Cell Counting Kit-8 assay. OD, optical density (n = 3). [file 13075_2021_2512_MOESM2_ESM.tif]

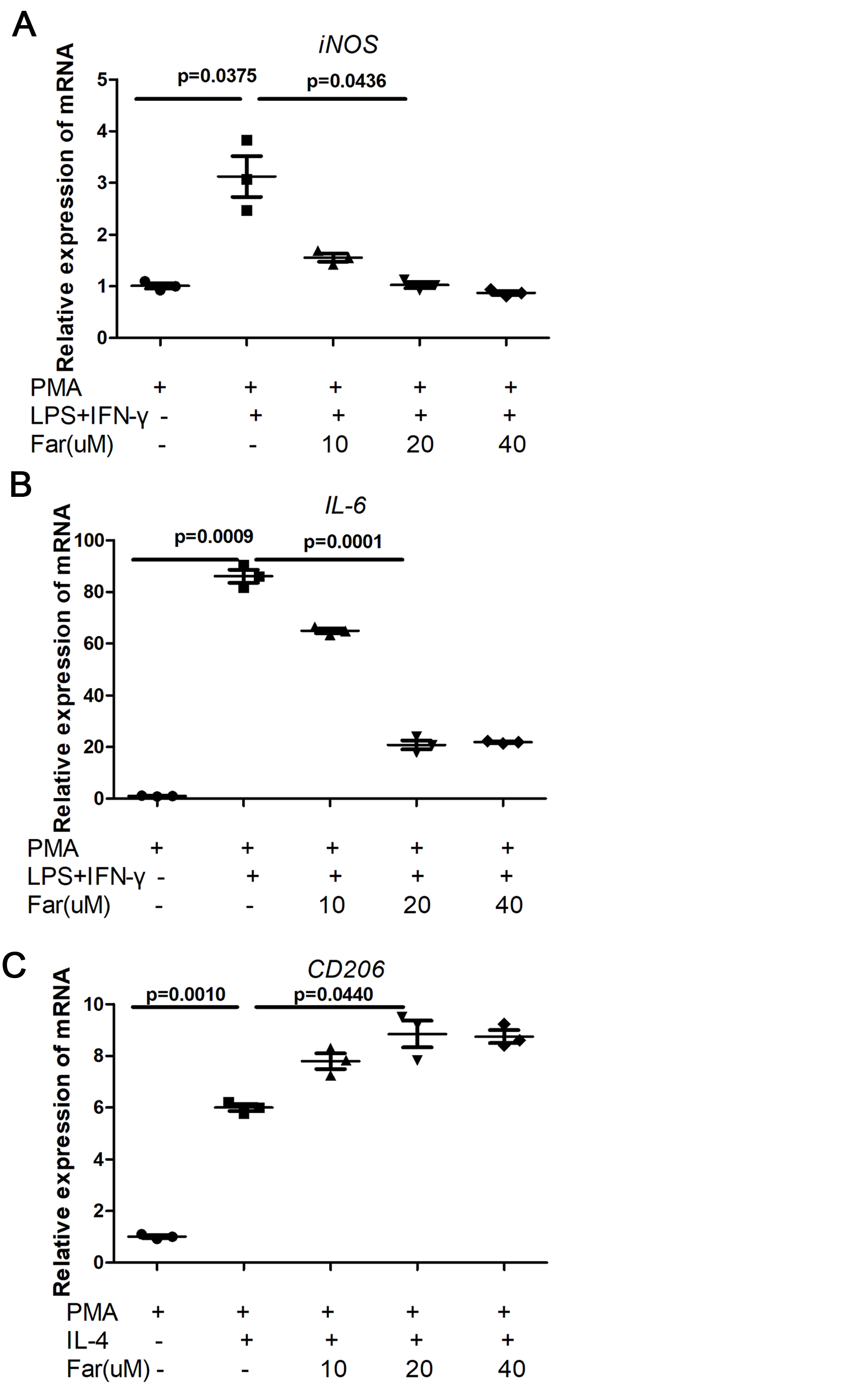

Supplement: Supplementary file 3 — Additional file 3: Figure S3. Fargesin acts as a potent polarizer towards M2 macrophages in THP-1 cells. (A–C) Quantitative PCR analysis of iNOS, IL-6, and CD206 in THP-1 cells treated with LPS or IL-4 and co-treated with vehicle or fargesin (n = 3). [file 13075_2021_2512_MOESM3_ESM.tif]

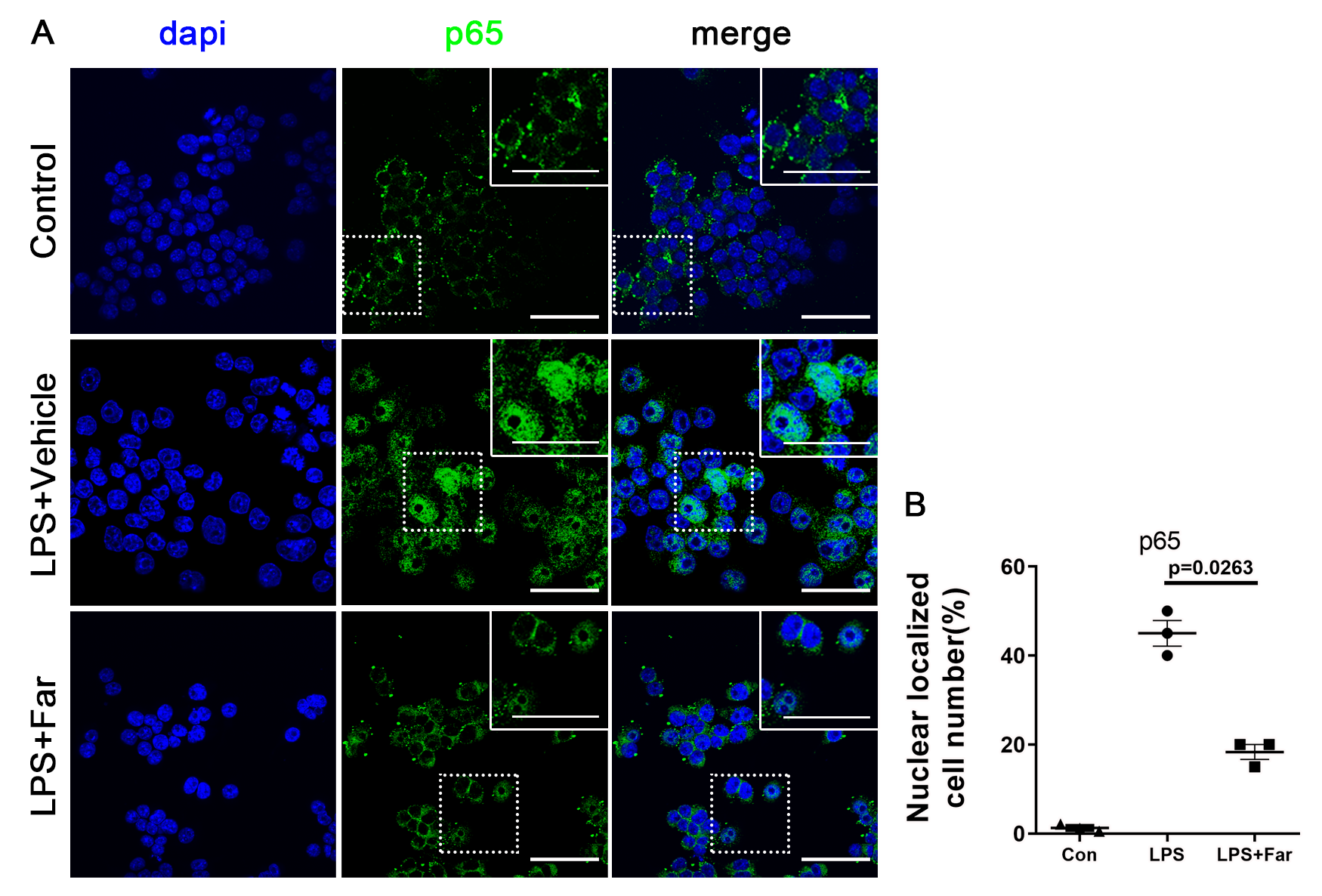

Supplement: Supplementary file 4 — Additional file 4: Figure S4. Fargesin treatment inhibits p65 nuclear translocation in LPS-induced Raw264.7 cell. (A and B) Immunostaining and quantitative analysis of nuclear localized cell for p65 in Raw264.7 cells treated with LPS (1 μg/mL) for 30 min after treated with vehicle or fargesin for 3 h. And control (Con) was treated without LPS (n = 3). Scale bar: 50 μm. Higher magnification is shown on the top right. Scale bar: 100 μm. [file 13075_2021_2512_MOESM4_ESM.tif]

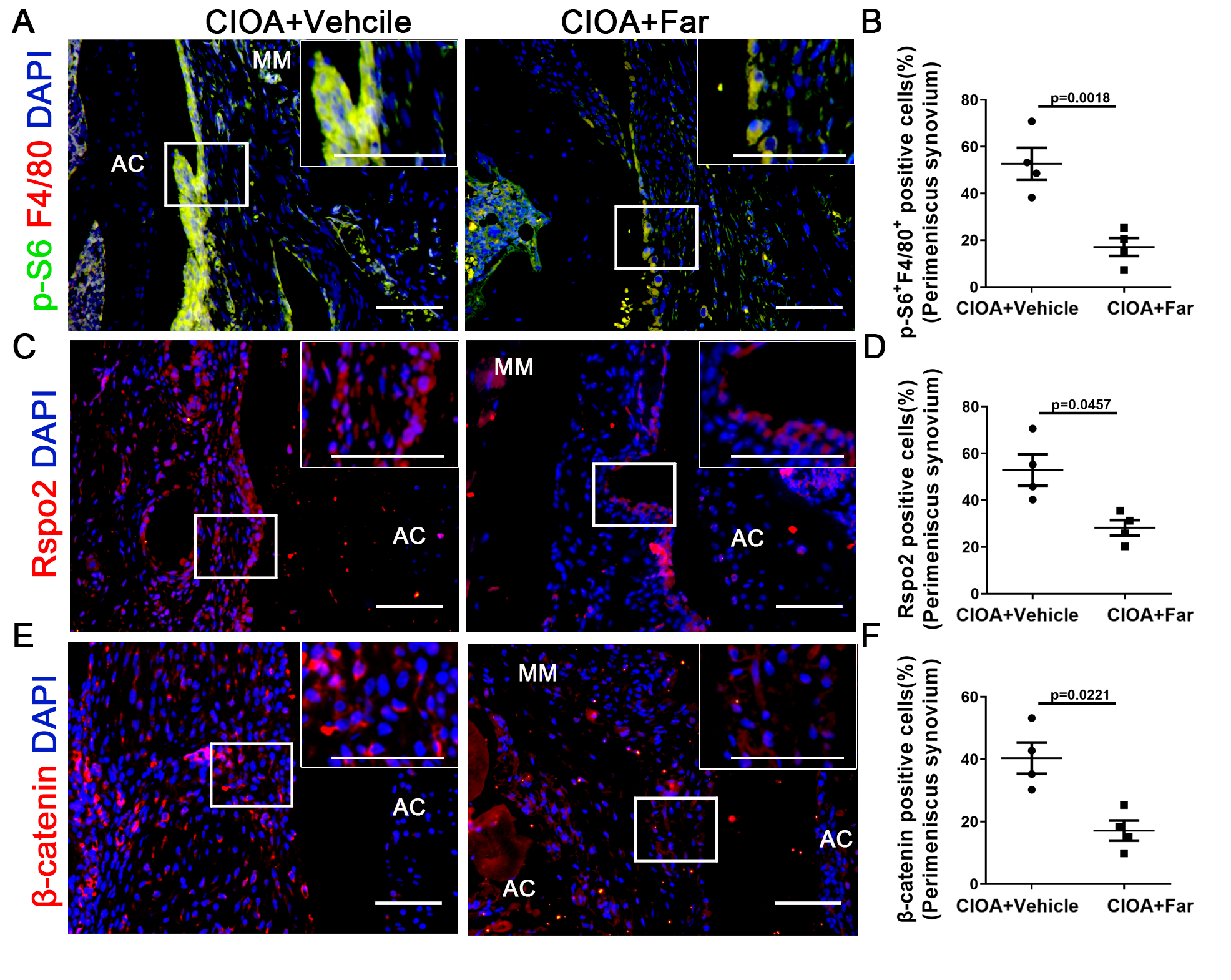

Supplement: Supplementary file 5 — Additional file 5: Figure S5. Fargesin attenuates p-S6, Rspo2 and β-catenin expression in synovial tissue of CIOA mice. (A-F) Immunostaining and quantitative analysis of cells positive for F4/80, p-S6 (A and B), Rspo2 (C and D), and β-catenin (E and F) in CIOA mice treated with vehicle or fargesin 3 weeks after intra-articular injection of collagenase. Scale bar: 50 μm. Higher magnification is shown on the top right. Scale bar: 100 μm. AC, articular cartilage; MM, medial meniscus. [file 13075_2021_2512_MOESM5_ESM.tif]

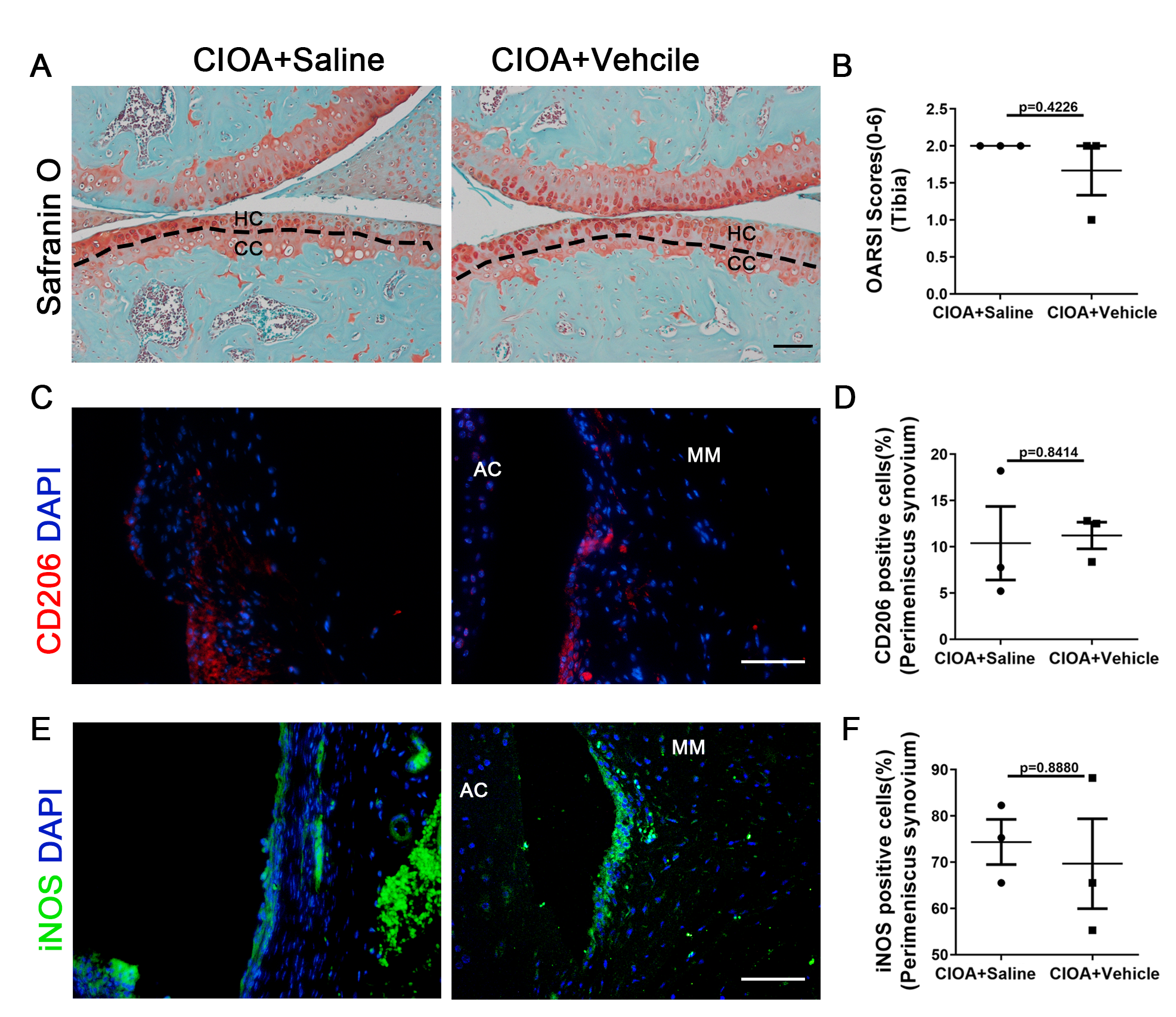

Supplement: Supplementary file 6 — Additional file 6: Figure S6. No significant improvement is observed in vehicle treatment compared to saline treatment 3 weeks after intra-articular injection of collagenase. (A) Cartilage degradation assessed by Safranin O and Fast Green staining. Dotted lines represent tide line. Scale bar: 50 μm (top). (B) OARSI score was evaluated in CIOA mice treated with saline or vehicle. (C-F) Immunostaining and quantitative analysis of cells positive for CD206 (C and D) and iNOS (E and F) in CIOA mice treated with saline or vehicle 3 weeks after intra-articular injection of collagenase. Scale bar: 50 μm. Scale bar: 25 μm. AC, articular cartilage; MM, medial meniscus. (n = 3). [file 13075_2021_2512_MOESM6_ESM.tif]
